# Translation and Psychometric Evaluation of the Greek Version of the Attitudes toward Transgendered Individuals Scale

**DOI:** 10.3390/bs14090739

**Published:** 2024-08-25

**Authors:** Dimitra Lekka, Argyro Pachi, Constantinos Togas, Athanasios Tselebis, Ilias Toliadis, George Alexias

**Affiliations:** 1 Psychiatric Department, Sotiria Thoracic Diseases Hospital of Athens, 11527 Athens, Greece; lekkadim@yahoo.gr (D.L.);; 2 Department of Psychology, Panteion University of Social & Political Sciences, Syggrou Ave. 136, 17671 Athens, Greece; 3 Department of Psychology, National and Kapodistrian University of Athens, 15784 Athens, Greece

**Keywords:** transgender people, transnegativity, stigma, attitudes, adjustment

## Abstract

Numerous investigations have consistently underscored the impact of societal stigma on the well-being of transgender individuals. The primary objective of the current research is to translate and adapt the Attitudes Toward Transgendered Individuals Scale into the Greek language. This scale specifically assesses stigma, excluding components such as discreteness and violence, and is tailored to evaluate individuals within the general populace. Employing confirmatory factor analysis (CFA) and assessing gender metric equivalence, the analyses yielded highly favorable outcomes, demonstrating excellent scale fit, reliability, and construct validity, reflecting the robustness of the adapted tool for the Greek population.

## 1. Introduction

Contemporary perspectives underscore the critical role of adverse attitudes towards transgender individuals in fostering the development of stigma against this demographic. The term “transgender” refers to individuals experiencing a dissonance between their self-identified gender and the sex assigned to them at birth [1]. The complexity of this definition is compounded by societal perceptions that often adhere to a binary understanding of gender and sex. Undoubtedly, the term “sex” pertains to the biological makeup of an organism (i.e., chromosomal composition), whereas “gender” encompasses the socially constructed perceptions of roles associated with each gender [2,3].

It is noteworthy that historically, transgender individuals were classified within the category of Gender Identity Disorders in previous editions of the *Diagnostic and Statistical Manual of Mental Disorders* (DSM). The DSM-5 refined this classification to “Gender Dysphoria,” emphasizing the distress stemming from the incongruity between experienced gender identity and biological sex [4]. Moving away from stigmatizing practices of psychiatrization of transgender individuals, according to the ICD-11 (*International Classification of Diseases*), gender incongruence is explicitly not a mental disorder [5]. Transgender stigma significantly impedes opportunities and access to essential resources across various domains, including employment and healthcare, thereby severely impacting this demographic’s physical and mental well-being [6]. Given the prevalent stigmatization of transgender individuals worldwide, often originating from entrenched gender stereotypes, this demographic contends with some of the highest rates of discrimination, bias, and violence among stigmatized groups [6,7,8,9]. Goffman (1986) described stigma as a defining attribute that diminishes social worth and distorts perceptions of individuals [10]. This pervasive force shapes attitudes and behaviors, perpetuating societal disparities [11]. Compared to previous years, Greece has achieved a significant increase in the protection of the rights of LGBTQIA+ individuals according to the annual ranking of European countries in relation to the rights of LGBTQIA+ people from the Rainbow map [12]. As reported in the Rainbow map data, both Estonia and Greece amended existing laws to allow same-sex couples to marry, while Greece also bridged gaps in anti-discrimination legislation to fully protect LGBTQIA+ people. With these changes, Greece managed to climb into 6th place in the rankings with a percentage of 70.77%, while last year it was in 16th place with a percentage of 56.7% [13].

Recent epidemiological data suggest that about 7 percent of adults identify as LGBTQIA+, according to an online survey conducted in 43 countries between April 2023 and March 2024 by Statista Consumer Insights [14]. The study found considerable variation between countries. The Philippines, the United States, and Israel had the highest percentage of people who identify as LGBTQIA+, with 11 percent each, while both Thailand and Canada occupy second position with 10 percent of adults, followed by Sweden, Brazil, and Australia, each with 9 percent. At the lower end of the spectrum, South Korea and Romania are present, with 3 percent of adults identifying as LGBTQIA+ individuals.

The stigma directed at transgender individuals manifests in a pervasive discrimination, a phenomenon known as transnegativity, which is defined as “any prejudicial attitude, discriminatory or victimizing behavioral action overtly or covertly directed toward an individual because they are, or are perceived to be, trans” [15]. It can be explicated through two primary lenses: the cognitive explanation and the dialogical psychological explanation. The cognitive perspective relates to group affiliations and the associated perceptions individuals are expected to hold, perpetuating transnegativity as a cognitive bias within certain societal groups. The dialogical psychological explanation operates on a societal level, encompassing three key elements: cultural and temporal variations in the conceptualization of transgender individuals, the emergence of transnegativity in response to shifts in the definition of gender, and the reactions of individuals to such changes that challenge established perspectives. Transnegativity, then, is thought to more accurately reflect the cognitive aspects associated with reactions, as opposed to the obsolete term transphobia, which is assumed to mostly encompass the affective response towards a transgender person [16].

The interconnection between transnegativity and homonegativity stems from prejudicial attitudes [17], and recent studies report that certain personality traits and ideological views are implicated [18,19]. Men, in particular, demonstrate higher levels of both homonegativity and transnegativity, and religious choices play a pivotal role in shaping these attitudes, as highlighted by Fisher et al. (2017) [20]. It is essential to recognize that attitudes towards transgender individuals are complex and influenced by various factors [21,22,23]. Tee and Hegarty’s (2006) UK survey disclosed that individuals with religious affiliations held more negative views toward transgender people, often aligning with conservative values and a binary gender perspective [24]. Stigma, as indicated by Poteat et al. (2013), profoundly affects transgender individuals, given their sexual orientation challenges societal norms regarding gender (sex) [25]. This leads to dual pressures—external psychological stress from societal expectations and internal conflict related to gender identity. Differentiating between transgender men and women, research reveals a notable disparity, with transgender women often facing more negative sentiments [26], although at times transgender men may confront additional marginalization [27]. Factors such as religious affiliation, an absolutist mindset, and resistance to gender equality, particularly towards women, contribute to these adverse attitudes. Additionally, limited exposure to diverse sexual identities is associated with negative attitudes towards transgender women. Heterosexual men with religious affiliations tend to exhibit more negative attitudes, whereas women with diverse sexual identities and lower religiosity show more positive sentiments [28]. Furthermore, negative attitudes are correlated with the desire to uphold patriarchal norms among men.

These findings underscore the imperative for comprehensive research utilizing validated instruments to measure attitudes towards transgender individuals within the Greek population. The current study aims to translate and psychometrically evaluate the Greek version of the Attitudes Toward Transgendered Individuals Scale (ATTI), offering valuable insights into societal attitudes within this context. In assessing societal attitudes towards LGBTQIA+ individuals, 83 measures have been developed to capture different dimensions of these attitudes, although several have been criticized for their weak attributes [29]. Notable scales include the Attitudes Towards Lesbian and Gay Men scale for both gay men and gay women by Herek (1994) [30], the Genderism and Transphobia Scale by Hill and Willoughby (2005) [16], the Transgender Attitudes and Beliefs Scale by Kanamori et al. (2017) [31], already adapted for the Greek population [32,33], and the Attitudes Toward Transgendered Individuals Scale by Walch et al. (2012) [34], which is presently employed to measure attitudes within the Greek population.

The efficacy of the Attitudes Toward Transgendered Individuals Scale (ATTI) in capturing people’s cognitive evaluations and affective reactions towards transgender individuals has been acknowledged in various studies and utilized in research on the prevalence and degree of stigma, discrimination, and prejudice directed at the transgender population [35,36,37,38]. The purpose of this self-report questionnaire is to collect data concerning the attitudinal and evaluative beliefs of the Greek population towards transgender individuals. Its primary objective is to explore whether the model aligns with the requisite psychometric properties indicative of a well-fitting assessment tool. This includes examining its unidimensionality and assessing its metric equivalence.

## 2. Materials and Methods

### 2.1. Measuring Tools

*Attitudes Toward Transgendered Individuals Scale*

The Attitudes Toward Transgendered Individuals Scale (ATTI) is a unidimensional metric gauging the attitudes of heterosexual individuals towards transgender persons, employing a 20-item Likert-type rating system (1 = strongly agree, 5 = strongly disagree). A “strongly disagree” response signals a positive attitude towards transgender individuals, with higher values denoting more favorable perspectives [34]. Notably, the scale encompasses individuals both pre- and post-surgery within the transgender category.

The translation of the ATTI into Greek was achieved using the translation–back-translation method proposed by Brislin (1970) [39]. In all analyses, items 1, 5, 8, 10, 12, 13, 14, 16, and 17 were reversed, as suggested by Walch et al. (2012) [34], to mitigate desirability response bias as outlined by Cronbach [40]. The original scale demonstrated high reliability with an alpha coefficient of 0.95, while within the specific sample, it exhibited a reliability coefficient of 0.93 (alpha = 0.93, lower = 0.93, upper 0.94). These coefficients surpass the threshold of 0.9, indicating high reliability.

*Greek Version of the Rosenberg Self-Esteem Scale*

We have used the Greek adaptation of the Rosenberg Self-Esteem Scale (RSES), which is a unidimensional scale designed to assess an individual’s self-esteem [41]. Comprising 10 items, participants rate their responses on a scale ranging from 1 (strongly agree) to 4 (strongly disagree). Notably, a response of “strongly disagree” indicates lower self-esteem, whereas “strongly agree” signifies higher self-esteem. The adapted scale demonstrates a reliability coefficient of alpha = 0.81 (0.79 with Guttman’s split-half analysis) [41], while within our sample, the reliability coefficient is alpha = 0.79. The inclusion of this scale serves the purpose of assessing discriminant validity for the ATTI, so we expect the RSES to have no correlation with the ATTI, as was the case in Walch’s et al. (2012) original research [34]. The same method to identify discriminant validity was employed in a recent relevant scale adaptation [17].

*Attitudes Toward Lesbians and Gay Men scale*

The Attitudes Toward Lesbians and Gay Men scale (ATLG) assesses heterosexual individuals’ attitudes toward gay individuals. Consisting of 20 questions, participants respond using a graduated scale where 1 signifies “strongly disagree” and 5 signifies “strongly agree” [42]. Higher values on the scale denote a more negative attitude toward homosexual individuals, while lower values indicate a more positive attitude. Grigoropoulos et al. (2010) validated the scale, reporting a reliability index of alpha = 0.91 [43]. In the specific sample analyzed, the reliability index was alpha = 0.95. This instrument was included for assessing the ATTI’s convergent validity, and thus we expect the two scales to be correlated.

### 2.2. Sample

As a standard procedure, before performing the adaptation of the ATTI, we conducted a pilot study with 176 participants from the Greek population to ensure that the scale’s items are all applicable Greek attitudes toward transgender individuals. Convenience sampling was employed for sample selection, comprising of individuals readily available and accessible during the survey period, conducted from 1 January 2019 to 31 December 2019. A total of 1796 participants from diverse regions across Greece were included, having been approached at their workplaces and residences. Among them, 1086 were women and 709 were men. Furthermore, demographic information regarding participants’ birthplace and current residence was collected and is presented in Table 1.

The average age of the sample is 35.38 years, with a standard deviation (SD) of 14.06. When examining age distribution by gender, women have an average age of 36.81, while men have an average age of 34.45. Regarding religious affiliation, the majority of participants identified as Orthodox Christians, constituting 81.4% of the sample. Concerning economic classification, the predominant proportions were affiliated with the middle class (54%) and the lower-medium class (40.1%).

### 2.3. Statistical Analyses

Data analysis was conducted using R software (version 4.0.1) [44]. To assess the factorial structure of the scale within the Greek population, confirmatory factor analysis (CFA) was employed (using the statistical package lavaan), supplemented by the U3 index to assess participant adaptation (using the PerFit package). The mvnormal Test package was used for Mardia’s test and the psych package for reliability analysis. Furthermore, the measurement invariance of the model across genders was evaluated. Finally, potential gender differences in latent variable means were explored. The original scale demonstrated high reliability, with a Cronbach’s alpha coefficient of 0.93.

## 3. Results

### 3.1. Reverse-Coded Items

Prior to the primary analysis, items with negative wording were subjected to reverse coding. Specifically, the reversed-coded items include Item 1, Item 5, Item 8, Item 10, Item 12, Item 13, Item 14, Item 16, and Item 17. The appropriateness of this reversal was verified using the Mardia test, which examines significance for kurtosis and skewness. The Mardia test results indicated violations of multivariate normality, with kurtosis = 8106.775 and skewness = 100.221, both yielding *p*-values < 0.001. Consequently, the weighted least squares mean and variance (WLSMV) algorithm was applied for analysis, chosen for its robustness when dealing with non-normally distributed categorical data, as recommended by Brown (2015) [45].

### 3.2. Assessment for Aberrant or Careless Responses

In this study, we also assessed for aberrant or careless responses, which are indicative of participants responding in a random or inattentive manner. As emphasized by Karabatsos (2003) [46], identifying individuals displaying such behaviors is crucial, given previous research suggesting that such responses can influence the psychometric properties of a scale, including model fit, as highlighted by Meijer and Sijtsma (2001) [47].

In our study, we utilized the U3 indicator [48] to detect individuals exhibiting random or inattentive responses, a method shown to be robust in identifying such behaviors [46]. U3 statistic is a valuable tool for identifying aberrations such as guessing, cheating, or other irregular response behavior. While the U3 indicator is primarily employed in item response theory models, its relevance extends to its potential impact on the fit of a confirmatory factor analysis model in the presence of deviant participants.

The initial fit of our original model, prior to outlier removal, indicated moderate-to-poor fit, with *χ*^2^ = 2666.134, df = 170, *p* < 0.001, RMSEA = 0.090, SRMR = 0.095, CFI = 0.95, and TLI = 0.94. The statistical significance of *χ*^2^ is acknowledged, considering its sensitivity to sample size. Other fit indicators, such as RMSEA exceeding 0.08 and SRMR surpassing 0.06 [49] suggested suboptimal fit, although CFI and TLI exceeded the 0.9 threshold.

To enhance fit, we examined modification indices, leading to the introduction of error (residual) correlations for six item pairs: I12:I14, I16:I17, I13:I14, I5:I14, I12:I13, and I8:I14. This refinement yielded improved fit indices for the model: *χ*^2^ = 1993.487, df = 162, *p* < 0.001, RMSEA = 0.079, SRMR = 0.82, CFI = 0.96, and TLI = 0.957. Despite the enhancement, the RMSEA remained marginal, nearing 0.08, and the SRMR did not exhibit a substantial improvement, remaining above 0.06. It is crucial to note that while modification indices guide adjustments, a theoretical basis for error correlations must also be considered in model refinement.

Subsequently, the U3 index was employed for multinomial models to detect and exclude outlier participants. The U3 values were computed, establishing a cut-off point of 0.3159 in this sample. According to this criterion, individuals with values exceeding this threshold were identified as deviant participants, resulting in a reduced sample size of the final group to 1551 individuals.

Upon removal of these outliers, the fit indices for the refined model demonstrated significant improvement (N = 1551): *χ*^2^ = 796.615, df = 170, *p* < 0.001, RMSEA = 0.049, SRMR = 0.055, CFI = 0.988, and TLI = 0.987. Notably, this improved fit was achieved without the need for modification indices, indicating that the initial marginal fit was influenced by the presence of these deviant participants. Furthermore, the omission of error correlations, which require a theoretical basis, was facilitated by the enhanced fit resulting from the removal of outliers.

### 3.3. Psychometrics of ATTI

Table 2 displays the standardized loadings of the items, all of which surpass the 0.4 threshold, affirming the strength of the relationships between the latent construct and the observed variables.

Lastly, the measurement invariance of the final model concerning gender was assessed. Metric equivalence in this context ensures that the model maintains the same meaning across different groups (i.e., between men and women) and evaluates the psychometric consistency between these groups [50]. This examination involves constraining certain parameters of the model to test the hypothesis of whether these parameters affect the fit of the model between groups.

For each parameter, various models were tested, including:

Configural model (initial): Verifying if the structure of the model is identical across genders.

Metric model: Checking if the factor loadings are equivalent across genders.

Scalar model: Assessing the equivalence of intercepts across genders.

Strict model: Examining if the errors (residuals) are equivalent across genders.

Model with constrained loadings, intercepts, errors, and latent means: Evaluating the overall equivalence. The results are presented in Table 3.

Applying the criteria of ΔCFI < 0.01 and ΔRMSEA < 0.015 [51,52], the analysis indicates both metric and scalar equivalence. The comparison of models considering pairwise differences reveals that the configural and metric models do not exhibit values surpassing those specified for the difference in RMSEA and CFI (specifically, a difference of 0.003 for CFI and 0.005 for RMSEA). Consequently, it is reasonable to assert that the model demonstrates an equivalent fit across genders, suggesting that any statistically significant differences are not attributed to varying perceptions of the questionnaire across genders.

This finding is further supported by the scalar model, where differences are also minimal, as well as the residual model, indicating that the overall fit of the questionnaire, inclusive of errors, remains consistent across genders. Regarding latent means, although the difference experienced a slight increase, no outliers were observed, affirming the model’s equivalence in terms of its construction.

Lastly, we examined the correlations between the RSES and ATTI, revealing a correlation of r = 0.05, *p* = 0.0371, while the correlation between the ATTI and ATLG was found to be r = −0.36, *p* < 0.001. Analyzing the final scores across genders, it was observed that men on average exhibit higher levels of transnegativity (M = 67.57, SD = 15.34) compared to women (M = 72.84, SD = 14.76). This distinction was statistically significant, as indicated by a *t*-test with Welch correction: t(1279.2) = −6.7514, *p* < 0.001.

## 4. Discussion

The present research aimed to evaluate the psychometric properties of the Attitudes Towards Transgendered Individuals Scale (ATTI) within the Greek population, given the limited number of surveys addressing individuals’ attitudes towards transgender people in the Greek context. A comprehensive assessment of validity was undertaken, encompassing both the factor structure and metric equivalence across genders.

The psychometric properties of the model demonstrated satisfactory outcomes, with factor analysis revealing good fit and robust loading, eliminating the necessity for question removal. Employing the U3 index to identify and exclude outliers resulted in a substantial improvement in the model’s performance. Additionally, our findings support the model’s one-dimensional structure, consistent with the framework proposed by Walch et al. (2012) [34]. The high reliability observed further affirms the robustness and adequacy of the model.

Regarding gender, we conducted an assessment of metric equivalence to determine if different groups perceive the concepts measured in the model differently. This is crucial, as disparities in understanding across genders could lead to methodological errors and spurious effects [53]. Our findings affirm that meaningful comparisons can be drawn between men and women concerning the final values derived from the questionnaire, alleviating concerns about gender-related disparities in question comprehension.

While it would have been beneficial to account for various demographic variables, the uneven distribution of groups prompted us to focus solely on gender as a covariate. This decision was influenced by the observed trend wherein men on average exhibited more negative sentiments towards transgender individuals compared to women. This aligns with previous research findings [34,54], reinforcing the satisfactory performance of the scale. Additionally, a moderate correlation (r= −0.36) emerged between transnegativity and homonegativity, as expressed by the correlation coefficient between the ATTI and ATLG. The negative correlation is a result of higher scores on the ATTI signifying less transnegativity and higher scores on the ATLG signifying more homonegativity. This correlation is consistent with previous research findings [32,54] and supports the convergent validity of the ATTI. The correlation of the ATTI with the RSES was also statistically significant, but negligible (r = 0.05), therefore supporting discriminant validity [17].

The present research is subject to several limitations. Firstly, the sample size was insufficient to enable examination of metric equivalence across selected demographics. While we assessed metric equivalence for gender, we could not extend this analysis to religious orientation or place of residence due to unequal sample sizes and disparities in subgroup representation. Another important limitation concerns the fact that our battery of questionnaires did not include questions regarding participants’ sexual orientation, which of course could affect the attitudes assessed by the employed psychometric tools. Moreover, there was a lack of data pertaining to convergent and discriminant validity, as the only scales included were the ATLG and RSES. Future studies could address this limitation by including more comparable questionnaires and exploring metric equivalence across diverse demographic groups. Also, an important drawback that has possibly influenced the content validity of the scale is that although a pilot study preceded its adaptation, we did not update the measurement tool to reflect current conventions.

Furthermore, it is worth noting the numerical disparity between men and women in our sample, which warrants further exploration in future studies. Additionally, implementing a test–retest method would be beneficial to evaluate the reliability of repeated measurements. As suggested by Walch et al. (2012) [34], future research could explore whether outcomes, such as positive or negative emotions toward transgender individuals, can predict attitudes toward this demographic group. Research regarding antitransgender attitudes and biased emotions and behaviors with the available psychometric instruments could explore if certain individuals’ basic beliefs and values can serve as predictors of transgender prejudice. The alarming rates of gender-based violence deeply impacting transgender and gender-diverse individuals [55] necessitate prevention and intervention strategies beyond legislative protection and compulsory modification of discriminatory and/or victimizing behaviors. Establishing protocols for early identification, monitoring, and addressing the prejudicial attitudes, primarily in educational institutions, is a major challenge [15].

Finally, we think it is very important to update the studies on the attitudes of healthcare staff toward transgender people. The pandemic caused an unintended, global economic recession while widening pre-pandemic health inequalities, social and economic, disproportionately affecting vulnerable populations by increasing their health needs [56,57], while at the same time, the pandemic put too much pressure on health workers [58,59]. In earlier studies before the pandemic crisis, health professionals showed favorable attitudes toward transgender people [60,61]. During the pandemic and immediately afterward, health workers showed aggression, anger [62,63], and cynicism [64], probably because of the pressure. However, this evidence may be consistent with an increase in negative attitudes towards populations now in greater health need [65].

## 5. Conclusions

Our findings suggest that the Greek version of the Attitudes Toward Transgendered Individuals Scale (ATTI) presents a coherent structure with high internal consistency and satisfactory convergent and discriminant validity. These results support the applicability of the Greek version of the ATTI within the Greek population, yielding equivalent fit across genders, and future studies could further explore the relevance of the scale with other measurement instruments and outcomes, its metric equivalence across other selected demographics, and its predictive validity.

## Figures and Tables

**Table 1:** Participants’ place of origin.

Place of Origin and Residence	Percentage of Origin	Rate of Accommodation
Village	2.3%	1.4%
Municipality	16.7%	12.5%
County capital	28%	27.4%
Athens/Thessaloniki	44.2%	55.8%
Island	4.6%	2.2%
Town	4.1%	0.8%
Total	100	100

**Table 2:** AΤΤΙ item loadings.

	Ν = 1551
Items	Loadings
Item 1: It would be beneficial to society to recognize transgenderism as normal.	0.73
Item 2: Transgendered individuals should not be allowed to work with children.	0.68
Item 3: Transgenderism is immoral.	0.78
Item 4: All transgender bars should be closed down.	0.65
Item 5: Transgendered individuals are a viable part of our society.	0.47
Item 6: Transgenderism is a sin.	0.69
Item 7: Transgenderism endangers the institution of the family.	0.74
Item 8: Transgendered individuals should be accepted completely into our society.	0.73
Item 9: Transgendered individuals should be barred from the teaching profession.	0.74
Item 10: There should be no restrictions on transgenderism.	0.57
Item 11: I avoid transgendered individuals whenever possible.	0.71
Item 12: I would feel comfortable working closely with a transgendered individual.	0.70
Item 13: I would enjoy attending social functions at which transgendered individuals were present.	0.71
Item 14: I would feel comfortable if I learned that my neighbor was a transgendered individual.	0.73
Item 15: Transgendered individuals should not be allowed to cross-dress in public.	0.66
Item 16: I would like to have friends who are transgendered individuals.	0.68
Item 17: I would feel comfortable if I learned that my best friend was a transgendered individual.	0.71
Item 18: I would feel uncomfortable if a close family member became romantically involved with a transgendered individual.	0.63
Item 19: Transgendered individuals are really just closeted gays.	0.57
Item 20: Romantic partners of transgendered individuals should seek psychological treatment.	0.61

Note: *p* < 0.001 for all loadings.

**Table 3:** Comparison of models for invariance measurement.

Ν = 1551 Model	*χ^2^*	df	*p*	RMSEA	SRMR	CFI	TLI	ΔCFI	ΔRMSEA	Crisis
Configural	796.615	170	<0.001	0.049	0.055	0.988	0.987	-	-	-
Metric	1102.749	359	<0.001	0.052	0.061	0.986	0.985	0.003	0.005	Metric–Configural
Scalar	1150.078	378	<0.001	0.051	0.062	0.985	0.985	0.001	0.001	Scalar–Metric
Residual	1205.716	398	<0.001	0.051	0.064	0.985	0.985	0.001	0.001	Residual–Scalar
Latent Means	1664.402	399	<0.001	0.064	0.074	0.976	0.977	0.009	0.013	Means–Residual

Note: Δ*CFI* = difference between models in terms of CFI, Δ*RMSEA* = difference of models in terms of RMSEA index.

## Data Availability

The data that support the findings of this study are available from the corresponding author, D.L., upon reasonable request.
